# Road traffic injury among elderly people and its determinant factors: a cross‐sectional study

**DOI:** 10.1002/hsr2.70098

**Published:** 2024-10-06

**Authors:** Saber Azami‐Aghdash, Naser Derakhshani, Riaz Alaei Kalajahi, Behrouz Samei, Ramin Rezapour

**Affiliations:** ^1^ Medical Philosophy and History Research Center Tabriz University of Medical Sciences Tabriz Iran; ^2^ Health Management and Economics Research Center, Health Management Research Institute Iran University of Medical Sciences Tehran Iran; ^3^ Student Research Committee Tabriz University of Medical Sciences Tabriz Iran; ^4^ Tabriz Health Services Management Research Center Tabriz University of Medical Sciences Tabriz Iran

**Keywords:** elderly, epidemiology, injuries, road traffic accident

## Abstract

**Background and aims:**

Injuries from Road Traffic Accidents (RTAs), particularly among vulnerable groups such as the elderly, are considered a significant public health concern. The aim of this study was to show epidemiology of RTIs among the elderly people in the Eastern Azerbaijan province of Iran.

**Method:**

This cross‐sectional study included all patients over 60 years old who admitted to the Shohada Hospital from 2006 to 2016. Data were extracted from the Hospital Information System. Injuries types were classified based on the International Classification of Diseases (ICD‐10). Descriptive statistics (Mean, median and frequency) and inferential statistics (The Pearson Chi‐square test) were used for data analysis through SPSS‐24 software.

**Results:**

A total of 3509 RTAs involving patients over 60 years old from 2006 to 2016. These traffic‐related injuries comprised 15% of all recorded injuries (23,321 cases). The mean age of the patients was 69.91 ± 7.61 years, with a predominant male representation (2324 patients, 64.74%). The median Length of Stay (LOS) in hospital was 3 days. Injuries most commonly affected the knee and lower leg regions (27.5%). The most frequent types of accidents were those involving car passengers (40.47%) and pedestrians (36.33%). The majority of RTAs occurred during the summer season. There was a significant difference in the rate of RTAs based on variables such as sex, age, season, and LOS (*p* < 0.001).

**Conclusion:**

The study highlights traffic‐related injuries among the elderly, mostly affecting the knee and lower leg regions, especially during the summer season. The efforts should include enhancing safety measures for car passengers and pedestrians, particularly during the high‐risk summer months. Also, it emphasizes the need for public transportation to improve safety for older people due to lower accident rates and senior‐friendly features.

## 1 INTRODUCTION

Road Traffic Accidents (RTAs) are cited as a leading cause of mortality and disability.^1^, ^2^ In 2021, it is estimated that 1.19 million individuals lost their lives on the roads, with 92% of these deaths occurring in low‐ and middle‐income countries.^3^ By 2030, Road Traffic Injuries (RTIs) are expected to rise from their present ranking as the eighth most common cause of mortality worldwide across all age groups to the seventh rank.^4^, ^5^, ^6^ Subsequently, the Millennium Development Goals monitors illustrate a 24% share for RTAs from the entire deaths due to different types of accidents.^7^ Furthermore, traffic crashes constitute the first death factor among groups aged 5–29 all across the world.^3^

Elderly people are among the groups at highest risk for injuries and accidents.^8^ The severity of harm and damage from these accidents is exacerbated in seniors due to diminished physical capabilities and increased susceptibility to physical distortions.^9^, ^10^, ^11^, ^12^ RTAs, in particular, have the highest incidence rates, mortality rates, and contribute significantly to decreased disability‐adjusted life years among the elderly.^13^, ^14^ Additionally, research indicates that over 35% of seniors who sustain injuries from accidents and seek hospital treatment are involved in RTAs.^14^

Injuries are the fifth leading cause of mortality among individuals aged 65 and older. Research suggests that the economic burden associated with the higher incidence rates of accidents in this demographic represents a substantial proportion of overall hospital expenditures.^14^, ^15^, ^16^, ^17^ This concern is amplified by the projected 1.6% increase in the global population of individuals over 60 years old.^18^ Additionally, it is anticipated that the elderly population will exceed 2 billion by 2025, a significant rise from the previously estimated 900 million in 2015.^19^ By 2050, it is expected that 80% of the elderly population will reside in low‐ and middle‐income countries.^20^ This demographic shift poses a substantial challenge to healthcare systems, including Iran's. In 2016, the elderly population constituted approximately 8% of the total population of Iran; however, this proportion is projected to increase to 12% by 2025^21^ and 26% within the next 40 years.^20^

A comprehensive study of the epidemiological patterns of RTIs among the elderly is crucial. With detailed data, policymakers can develop effective preventive measures and strategies. Therefore, the aim of this study was to investigate the epidemiology of RTIs among the elderly population who admitted to Shohada Hospital in Eastern Azerbaijan Province from 2006 to 2016.

## 2 METHOD

### 2.1 Study type and population

This cross‐sectional study included all patients aged 60 and older who admitted to the Shohada Hospital from 2006 to 2016. The hospital, which is a leading provider of orthopedic services in northwestern Iran, also serves as a referral center for trauma cases, with 265 active patient beds.

### 2.2 Data collection

A self‐constructed form was used to collect data on demographics information (Age, sex), type of the RTAs, type of RTIs, the accident time, and Length of Stay (LOS) in hospital. All information was extracted from the Hospital Information System (HIS).

### 2.3 Data analysis

The ICD‐10 international classification system^22^ was utilized to categorize the types of road users and the injuries (Tables 1 and 2). This coding system encapsulates a vast amount of detailed information about diseases and injuries into specific codes, such as S01.2. To facilitate analysis process, data were cleaned and organized. Initially, the codes from the HIS were classified based on the ICD‐10 system (Tables 1 and 2). Subsequently, these codes were assigned numerical values from 1 to 10 (e.g., 1 = S00–S09, 10 = S90–S99).

**Table 1:** Classification of road traffic injuries based on ICD‐10.

Items	ICD‐10 code	Injury type
1	S00–S09	Injuries to the head
2	S10–S19	Injuries to the neck
3	S20–S29	Injuries to the thorax
4	S30–S39	Injuries to the abdomen, lower back, lum…
5	S40–S49	Injuries to the shoulder and upper arm
6	S50–S59	Injuries to the elbow and forearm
7	S60–S69	Injuries to the wrist, hand and fingers
8	S70–S79	Injuries to the hip and thigh
9	S80–S89	Injuries to the knee and lower leg
10	S90–S99	Injuries to the ankle and foot

**Table 2:** Classification of road users' types injured in road traffic accidents based on ICD‐10.

Items	ICD‐10 code	User type
1	V00–V09	Pedestrian injured in transport accident
2	V10–V19	Pedal cycle rider injured in transport accident
3	V20–V29	Motorcycle rider injured in transport accident
4	V30–V39	Occupant of three‐wheeled motor vehicle injured in transport accident
5	V40–V49	Car occupant injured in transport accident
6	V50–V59	Occupant of pick‐up truck or van injured in transport accident
7	V60–V69	Occupant of heavy transport vehicle injured in transport accident
8	V70–V79	Bus occupant injured in transport accident
9	V80–V89	Other land transport accidents
10	V90–V94	Water transport accidents
11	V95–V97	Air and space transport accidents
12	V98–V99	Other and unspecified transport accidents

Age groups were divided into three subgroups—60 to 74, 75 to 89, and 90 years and older—based on the World Health Organization's age categorization format.^23^ Regarding the “length of stay,” the median (3 days), first quartile (1 day), and third quartile (6 days) were calculated. Consequently, this variable was divided into four subgroups: “1 day or less,” “two to 3 days,” “four to 6 days,” and “7 days or more.”

Descriptive statistics including frequency, relative frequency, median, mean, and standard deviation were employed to describe RTAs types, RTIs types and other demographics variables. Also, Pearson's Chi‐square test was applied to assess the difference in relative frequency of RTAs and RTIs within gender groups, age groups, seasons, and multiple categories of the LOS. Significance level was obtained lower than 0.05 (2‐tailed) and SPSS‐24 software was used to data analysis.

### 2.4 ETHICS APPROVAL

This study was part of an approved study in the Research Ethics Committee of Tabriz University of Medical Science (ethical code: IR. TBZMED.REC.1398.139). Methods were performed in accordance with the relevant guidelines and regulations. All authors have read and approved the final version of the manuscript. [Ramin Rezapour] have full access to all of the data in this study and take complete responsibility for the integrity of the data and the accuracy of the data analysis.

## 3 RESULTS

A total of 3509 RTAs involving individuals aged over 60 were recorded from 2006 to 2016, accounting for 15% of all RTIs (23,321 elderly patient). The mean age of the population was 69.91 ± 7.61 years. The majority of the injured were male (2324 elders or 64.74%), and the median LOS was 3 days. Injuries to the knee and lower leg were the most common, occurring in 27.5% of cases, while neck injuries (0.4%) and thoracic injuries (0.5%) were rare (Table 3).

**Table 3:** The rate of road traffic injuries among elderly people, 2006–2016.

Type of injury	Frequency	Relative frequency (%)
Injuries to the head	236	7.0
Injuries to the neck	13	0.4
Injuries to the thorax	17	0.5
Injuries to the abdomen, lower back, lum…	267	8.0
Injuries to the shoulder and upper arm	412	12.3
Injuries to the elbow and forearm	429	12.8
Injuries to the wrist, hand and fingers	275	8.2
Injuries to the hip and thigh	560	16.7
Injuries to the knee and lower leg	923	27.5
Injuries to the ankle and foot	220	6.6
Total	3352	100.0

**Figure 1:**
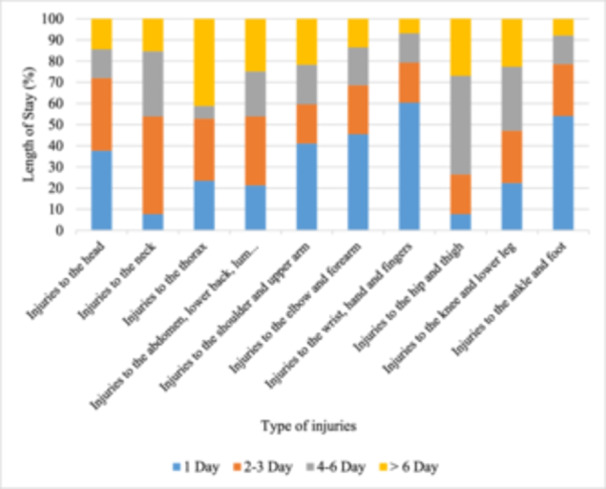
Elderly people road traffic injuries based on length of stay in hospital.

The provided data (Figure 1) depicts the distribution of elderly people's RTIs across different body regions, segmented by the LOS in hospital: 1 day, 2–3 days, 4–6 days, and more than 6 days. The data highlights that while some injuries among elderly road traffic victims are minor and treated quickly, others, particularly thoracic and hip/thigh injuries, often require prolonged hospital stays due to their severity.

**Figure 2:**
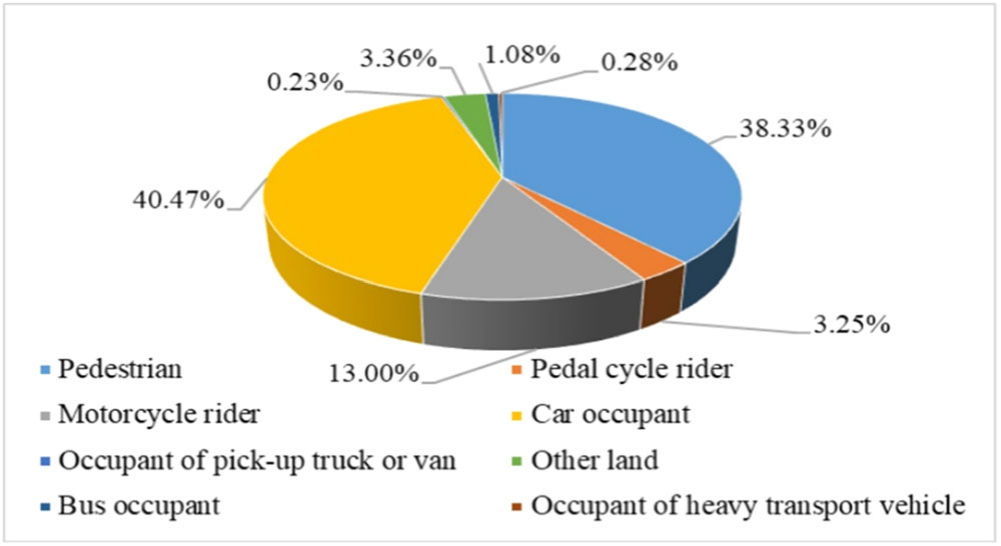
Types of users injured in road traffic accidents, 2006–2016.

Additionally, car occupants, pedestrians, and motorcycle riders were the most frequently injured, with incidences of 40.47%, 36.33%, and 13%, respectively. In contrast, occupants of pickup trucks or vans and heavy transport vehicles had the lowest relative frequencies, accounting for only 0.23% and 0.28% of the total injuries, respectively (Figure 2). No records were available for uncommon accidents involving tricycles, aerospace, maritime transportation, or other related incidents.

**Figure 3:**
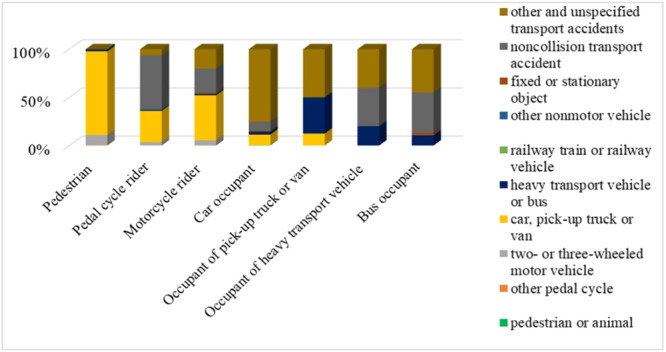
Distribution of road traffic accidents among different user types.

As shown in Figure 3, the ICD‐10 classification provides subgroups for each type of road traffic users, identifying the type of vehicle involved. According to these subgroups, unspecified transport accidents are prevalent across all categories. For pedestrians, the most common accident was being hit by a car, pickup truck, or van, accounting for 86.84% of incidents. Pedal cycle riders were primarily injured in non‐collision transport accidents (56.14%), while motorcycle riders were most frequently hit by cars, pickup trucks, or vans (46.71%).

**Figure 4:**
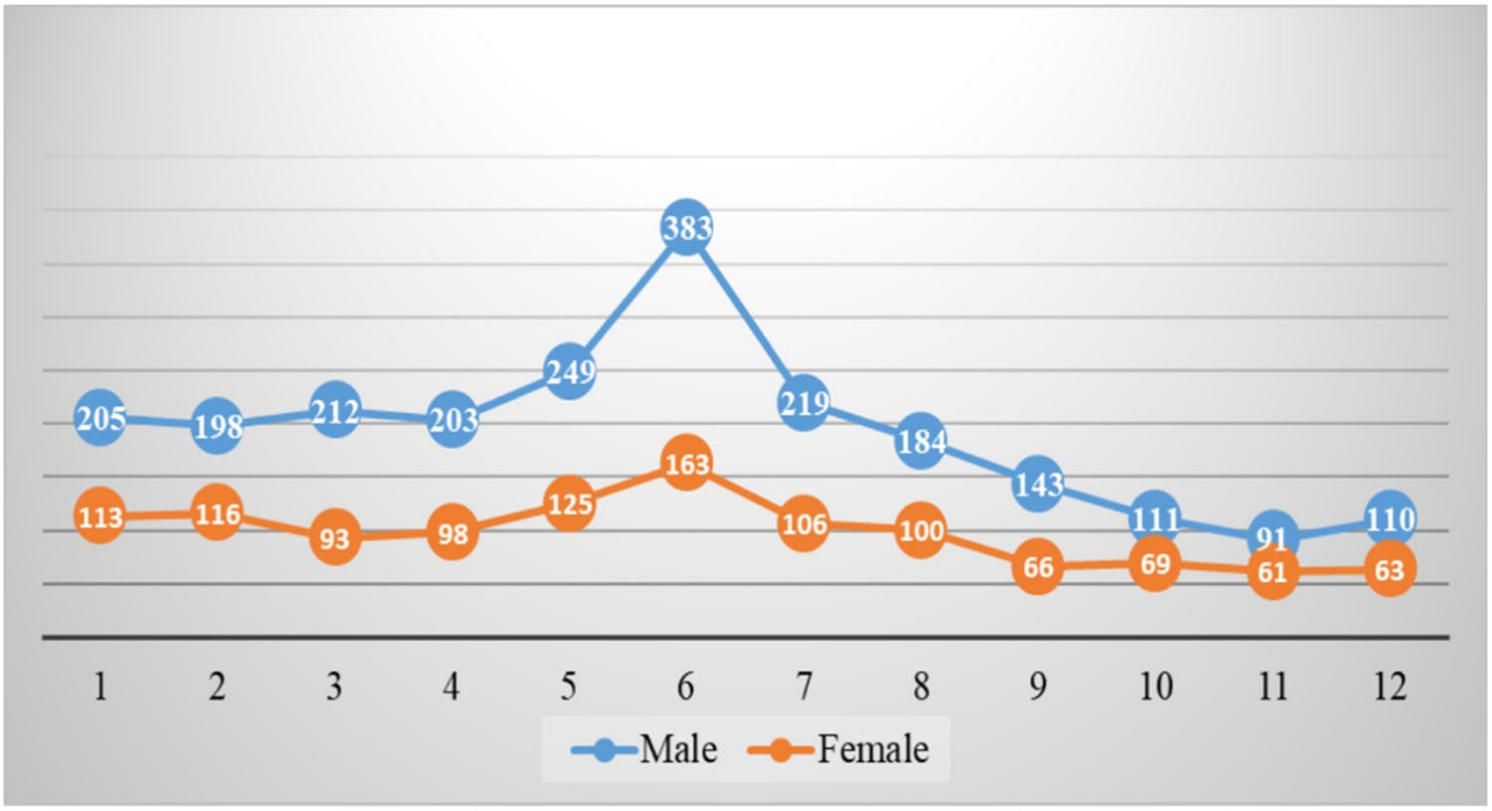
A gender‐based monthly distribution of traffic accidents over a period from 2006 to 2016.

The Figure 4 illustrates the monthly distribution of RTAs among males and females over a period from 2006 to 2016. There were significant differences in the frequency of RTAs across different months for both genders (*p* < 0.001). The data reveals that the total number of RTAs involving males is significantly higher than those involving females. Specifically, males experienced 2408 RTAs, while females experienced 1173 RTAs over the same period. This indicates that males had more than twice the number of RTAs compared to females.

There is a significant spike in RTAs for both genders during the summer months, particularly in June. A noticeable decline in RTAs is observed during the winter months, with the lowest points occurring in October.

Pearson's Chi‐square test results indicated a significant difference in the frequency of RTAs across various groups based on age, gender, time (season or month), and LOS (*p* < 0.001) (Table 4). The data revealed that males were predominantly affected, with 2235 cases. The most impacted age group was those between 60 and 74 years old, accounting for 2454 cases. Interestingly, the highest number of RTAs occurred during the summer season, with a total of 1167 cases. Additionally, a significant number of patients, 1074, were discharged after just a one‐night stay in the hospital.

**Table 4:** Affecting factors on Traffic Accidents types.

Variables		Type of Traffic Accidents (*N*)							Total	*P* value*
		Pedestrian	Pedal cycle rider	Motorcycle rider	Car occupant	Occupant of pick‐up truck or van	Occupant of heavy transport vehicle	Bus occupant		
Sex	Male	844	101	382	876	6	7	19	2235	*p* > 0.001
	Female	501	13	74	544	2	3	19	1156	
Age	60–74	886	75	368	1082	7	8	28	2454	*p* > 0.001
	75–89	442	39	85	329	1	2	9	907	
	90<	17	0	3	9	0	0	1	30	
Season	Spring	335	32	118	396	1	7	12	907	*p* > 0.001
	Summer	444	37	192	479	5	1	9	1167	
	Autumn	345	28	89	320	2	2	10	796	
	Winter	209	14	56	213	0	0	7	499	
LOS#	1 Day	354	32	146	524	2	1	15	1074	*p* > 0.001
	2–3 Day	347	30	110	297	2	2	4	792	
	4–6 Day	377	34	110	310	2	4	9	846	
	>6 Day	267	18	90	288	2	3	10	678	

^#^ Length of Stay.

## 4 DISCUSSION

RTAs accounted for 15% of all accidents involving the elderly. The most common injury was to the knee and lower leg, representing 27.5% of cases. Care occupants and pedestrians were the most frequently injured groups, with shares of 40.47% and 36.33%, respectively. Most of these RTAs occurred during the summer season.

The study results showed that a significant percentage (15%) of accidents affecting elderly people were traffic‐related. The incidence of RTAs among the elderly shows considerable variation across different studies. In the Federal District of Brazil, research documented 249 RTAs death involving the elderly over a 5‐year span, with a higher prevalence among men aged 60–69, primarily due to trampling and collisions.^8^ In Iran, another study revealed that RTIs made up 23.6% of total injuries among the elderly, with car accidents and pedestrian injuries being the most common. This study also noted significant differences in mortality rates between elderly and non‐elderly individuals.^24^ Furthermore, research in a Brazilian state found that older car drivers had a lower crash risk compared to adult drivers, particularly on urban, low‐speed streets, although there was a smaller decrease in crash incidence over time.^25^ According to additional research, young drivers aged 15–24 are more likely to be involved in RTAs due to their lack of experience and tendency to ride with inexperienced drivers.^26^ Conversely, elderly drivers also show higher rates of RTAs and a greater incidence of injuries compared to younger drivers.^27^ Furthermore, individuals aged 60 and above involved in motor vehicle accidents exhibit distinct injury patterns and are less resilient to sustained injuries, resulting in a higher mortality rate when compared to younger adults.^28^ These findings highlight the need to understand and address the specific patterns and risks of RTAs among the elderly to enhance road safety and reduce mortality rates.

Additionally, the most frequent injuries involved the knee and lower leg. This result contrasts with the findings of numerous other epidemiological studies in the same field. Some studies have shown that facial and head injuries play a significant role in causing injuries and fatalities in RTAs.^29^, ^30^, ^31^ In contrast, some studies have reported higher rates of lower body injuries among elderly individuals who were injured or died due to RTAs.^32^, ^33^ This is particularly highlighted by a systematic review from 2018, which found that impacts to the head, face, and lower body were the leading factors causing deaths and disabilities in RTAs.^34^ Therefore, while general preventive measures are important, it is highly recommended to implement specific and targeted interventions to reduce the risk and likelihood of impacts to these areas.

Furthermore, pedestrians and car occupants were the two groups most frequently involved in RTAs. Many studies, including those by Nagata et al. in Japan,^35^ Bhalla et al. in Iran,^36^ Shuai et al. in China,^31^ Richter et al. in Germany,^37^ and Saveman and Bjornstig in Sweden,^38^ have identified elderly car occupants as the primary group injured in these accidents. This highlights the crucial importance of considering the safety of pedestrians and car occupants in the design and implementation of traffic policies. Pedestrians are particularly vulnerable to serious injury or death when struck by vehicles due to their lack of physical protection. Similarly, car occupants are at an elevated risk, especially those in older vehicles with potentially lower safety standards. Consequently, enhancing urban infrastructure for pedestrians, such as by constructing safe sidewalks and crosswalks, along with improving vehicle safety standards, can significantly reduce crash rates and the severity of injuries.

Unexpectedly, a significant proportion of elderly individuals (13%) were injured while riding motorcycles. Studies conducted in Egypt^39^ and Australia^40^ reported accident rates of 0.7% and 0.5%, respectively. Remarkably, Etehad et al.^30^ identified even higher accident rates (19.1%) among elderly motorcyclists. The socioeconomic vulnerability of senior citizens likely contributes to their reliance on motorcycles due to financial constraints preventing car ownership. In recent years, there has been a notable surge in motor vehicle production, particularly motorcycles, with over 60 active mass‐production factories in Iran. However, this increase has not been accompanied by a commensurate advancement in the cultural understanding of safe motorcycle usage, highlighting a significant gap in public awareness regarding safe riding practices.^41^ Given motorcycles’ limited safety features for riders,^42^, ^43^ advocating for safer design standards, stringent legislative measures, and targeted interventions to promote helmet use represent crucial strategies for mitigating this issue.

Several studies have indicated a notable increase in RTAs during the summer season in Iran, which corroborates earlier findings.^44^, ^45^, ^46^, ^47^, ^48^ Considering trips happening at this time, especially at the end of summer (August 20 till September 20), it is predictable to have a sudden or even a sharp increase in RTAs rates. Similarly, statistics reveal a parallel rise in accidents during the spring holiday season (March 20 to April 20). While it is crucial for elderly drivers to exercise vigilance to mitigate RTAs risks, it remains imperative for law enforcement, notably the police, to implement stringent monitoring of traffic behaviors. A significant consideration is the psychological and physiological factors influencing these trends. Elderly drivers, especially during these periods, exhibit higher risk‐taking behaviors, potentially exacerbated by seasonal weather changes. Spring brings frequent rain showers, while summer sees warmer temperatures leading to driver fatigue and increased RTAs rates.

## 5 CONCLUSION

This study highlights key areas of concern about RTIs among the elderly. These RTIs mostly affected the knee and lower leg regions, and were most common among car passengers and pedestrians. The summer season emerged as a particularly high‐risk period for RTAs. Additionally, significant differences in RTA rates were observed across various demographic and seasonal variables, emphasizing the need for targeted interventions. The elderly population's safety can be greatly improved by addressing these particular risks through customized preventive measures.

Additionally, it is essential to consider the function of public transit in improving safety for older people. Public transportation, particularly buses, is safer for older people than private vehicles because of lower accident rates, improved crash safety, and senior‐friendly features.

## 6 LIMITATION

The study provides valuable insights by gathering information over a 10‐year period. However, there is a limitation due to data incompleteness. Since the data were not originally collected for epidemiological research purposes, experts and researchers face significant challenges when analyzing them. To address this issue, it is recommended that future data collection efforts use prospective and standardized approaches.

## CONSENT FOR PUBLICATION

Not applicable in this section.

## AUTHOR CONTRIBUTIONS

**Saber Azami‐Aghdash**: Writing—original draft; methodology; software. **Naser Derakhshani**: Methodology; formal analysis. **Riaz Alaei Kalajahi**: Writing—original draft; methodology; data curation. **Behrouz Samei**: Writing—original draft; methodology; software. **Ramin Rezapour**: Writing—original draft; methodology; data curation; project administration.

## CONFLICT OF INTEREST STATEMENT

There is no conflict of interest relevant to this article.

## TRANSPARENCY STATEMENT

The lead author Ramin Rezapour affirms that this manuscript is an honest, accurate, and transparent account of the study being reported; that no important aspects of the study have been omitted; and that any discrepancies from the study as planned (and, if relevant, registered) have been explained.

## Data Availability

The data used in this article were obtained with the permission of Tabriz University of Medical Sciences. In case of request, the data can be accessed by the corresponding author: rrezapour313@gmail.com after obtaining permission to republish the data from Tabriz University of Medical Sciences.
